# Associations of sleep disturbance and emotion regulation with perceived workplace safety and fatigue among male firefighters with prior occupational accidents: A cross-sectional study

**DOI:** 10.1097/MD.0000000000051154

**Published:** 2026-10-23

**Authors:** Ahmet Erhan Bakirci

**Affiliations:** a Department of Civil Defense and Firefighting, Vocational School of Technical Sciences, Istanbul University-Cerrahpasa, Istanbul, Türkiye.

**Keywords:** cross-sectional studies, emotion regulation, firefighters, occupational fatigue, perceived workplace safety, sleep disturbance

## Abstract

Firefighters bear a high burden of sleep disturbance and occupational accidents, yet how their individual health states relate to their own appraisal of workplace safety and fatigue is unclear. This cross-sectional study examined whether sleep disturbance, sleep quality, and emotion-regulation capacity are associated with perceived workplace safety and fatigue-related status in this group. One hundred five active-duty male firefighters from urban brigades in the Marmara region of Türkiye, all with a documented occupational accident history, completed the Basic Scale on Insomnia Complaints and Quality of Sleep, a single-item Sleep Quality Scale, the Emotion Regulation Skills Questionnaire, and the Perceived Workplace Safety/Fatigue Status tool (PWSFS), an exploratory, author-developed composite scored 20 to 100, with higher scores being more favorable (Cronbach α = 0.71 in this sample). Pearson correlations and a multiple linear regression of the PWSFS total score on the 3 predictors, adjusted for age and fire-service experience, were computed. The mean Basic Scale on Insomnia Complaints and Quality of Sleep was 12.2 (standard deviation 5.0), and the mean PWSFS was 73.2 (standard deviation 11.2). Greater sleep disturbance was associated with less favorable perceived safety and fatigue status (*r* = −.81, 95% confidence interval − .87 to − .74, *P* < .001), whereas better sleep quality (*r* = .52, *P* < .001) and greater emotion-regulation skills (*r* = .44, *P* < .001) showed weaker associations. The model accounted for 67% of the variance (F(5, 99) = 39.74, *P* < .001), and sleep disturbance was the only unique correlate (β = −.81, *P* < .001); neither sleep quality (*P* = .46) nor emotion regulation (*P* = .47) contributed independently. The association persisted in reduced-content analyses but was attenuated when sleep- and fatigue-related items were removed (safety-awareness subset *r* = −.58, *R*^2^ = .38, β = −.77). Among firefighters with prior occupational accidents, sleep disturbance was the measured individual-level variable most strongly associated with perceived workplace safety and fatigue. Because the design was cross-sectional and relied on single-source self-report, and because the outcome was measured with an exploratory, author-developed composite, these associations are provisional and cannot support causal inference. Sleep may nonetheless warrant attention in occupational safety and fatigue-management efforts; longitudinal, multi-source research is needed to confirm these findings.

## 1. Introduction

Firefighting is among the most physically and psychologically demanding occupations, exposing personnel to unpredictable operational schedules, sustained physiological strain, and recurrent exposure to critical incidents.^[1,2]^ Alongside these chronic demands, firefighters bear a substantial burden of occupational accidents and injuries, which carry both physical and psychological consequences.^[3–5]^ Firefighters who have experienced such accidents are a group of particular interest for occupational-health surveillance, yet how their individual health states relate to their own appraisal of workplace safety and fatigue remains poorly understood.

The occupational hazard profile of this workforce extends well beyond psychological demand. In 2022 the International Agency for Research on Cancer classified occupational exposure as a firefighter as carcinogenic to humans, on the basis of sufficient evidence in humans for mesothelioma and bladder cancer.^[6]^ Polycyclic aromatic hydrocarbons and other products of incomplete combustion reach firefighters both by inhalation and through the skin during fire suppression, with measured concentrations rising as exposure duration increases.^[7]^ Simulated fire suppression on its own produces measurable heat strain and acute adverse cardiovascular effects in healthy firefighters.^[8]^ These lines of evidence document distinct hazards rather than a single common endpoint: the first concerns carcinogenic and chemical exposure, the second acute thermal and cardiovascular load. Their relevance to the present question is indirect, in that they establish a working environment in which several physiological burdens accumulate alongside the psychological ones. Evidence that occupational burden translates into reduced alertness and impaired cognitive performance comes from other high-exposure occupations, where occupational stress and the duration of underground work together accounted for close to 40% of the variance in cognitive performance among mine workers; that evidence is drawn from mining rather than firefighting and is therefore indirect for the present population.^[9]^ Within this profile, sleep disturbance occupies a particular position. It is not the largest hazard firefighters face, but it is one of the few that is routinely measurable in the field, plausibly downstream of several of the others, and modifiable through scheduling, recovery, and health-surveillance policy. That is the reason it is examined here, and not because the remaining exposures are less consequential.

How firefighters perceive the safety of their working environment, including safety awareness, fatigue management, health monitoring, and the maintenance of safety standards, is a practically important but comparatively under-examined dimension of occupational health.^[10,11]^ Firefighters’ safety attitudes, behaviors, and risk perceptions vary across operational and cultural contexts,^[12,13]^ and organizational safety culture has been proposed as a meaningful marker of workforce well-being.^[10,11]^ Individual affective and health states appear intertwined with such perceptions: safety climate and emotional exhaustion interact in shaping first responders’ safety behavior over time,^[14]^ and firefighters’ mental health has been linked to their risk- and safety-related perceptions.^[15]^ Two individual states are especially relevant to how safe and fatigue-resilient firefighters feel at work: disturbed sleep and the capacity to regulate emotions.

Sleep disturbance is highly prevalent in the fire service. A systematic review and meta-analysis reported a high pooled prevalence of poor sleep quality and sleep disorders among firefighters,^[16]^ and recent work suggests that restorative sleep may be difficult to achieve even away from duty,^[17]^ with elevated insomnia symptoms and excessive daytime sleepiness documented across national samples.^[18,19]^ Extended and irregular shift schedules and work-schedule instability disrupt circadian rhythms and are associated with poorer sleep and a higher risk of insomnia,^[20,21]^ while fluctuating emergency-call volume has been linked to greater occupational workload, poorer sleep quality, and altered perceptions of stress and recovery in active-duty firefighters.^[22,23]^ Disturbed sleep and accumulated fatigue have been associated with impaired alertness, diminished occupational performance, and health and safety consequences relevant to public-safety personnel.^[24,25]^ A dedicated cohort study examining firefighter work schedules and cardiovascular, well-being, and safety outcomes is currently in progress.^[26]^ Sleep disturbance in operational personnel is also heterogeneous, encompassing insufficient, fragmented, and poor-quality sleep rather than clinical insomnia alone.^[27,28]^

Emotion regulation, the capacity to monitor, evaluate, and modulate emotional responses, has emerged as a transdiagnostic correlate of mental-health outcomes in high-stress occupations.^[29,30]^ Among firefighters, emotion-regulation difficulties and related emotion-management processes have been associated with poorer psychological outcomes^[31,32]^ and with sleep-related risk, including associations between sleep disturbance and specific emotion-regulation dimensions.^[33]^ Heightened cognitive and emotional arousal is thought to interfere with sleep, and an intensive longitudinal study of emergency medical service clinicians who regularly use alcohol or cannabis found dynamic, bidirectional links between perceived stress and emotion regulation in a sample the authors note may not represent responders without regular substance use,^[34]^ suggesting that regulatory capacity and sleep are interrelated. The evidence is not uniform: in fire-service shift workers, self-reported sleep duration and efficiency predicted recovery-day stress and fatigue, whereas emotion-regulation variables did not, and neither acted as a mediator. Their temporal ordering, in any case, cannot be established in a cross-sectional design.^[35]^

Despite these parallel lines of work, the individual-difference tradition, which links sleep and emotion regulation to firefighters’ wellbeing, and the organizational tradition, which links safety climate and fatigue to safety behavior, have developed largely separately. Whether individual states such as disturbed sleep and emotion-regulation capacity are associated with firefighters’ own perceptions of workplace safety and fatigue-related status has received little direct empirical attention, particularly among firefighters with a documented history of occupational accidents. This gap is more pronounced in the Turkish occupational health and safety literature, which has remained predominantly descriptive, has drawn only sparingly on psychological variables such as safety climate, and has included comparatively little hypothesis testing.^[36]^ In a cross-sectional design, such an accident history is best understood as a sampling characteristic rather than a measured exposure, and its recency, severity, and consequences may vary across individuals.

The present cross-sectional study, therefore, examined active-duty male firefighters with a documented occupational accident history, with 3 aims: to describe sleep disturbance, sleep quality, emotion-regulation capacity, and perceived workplace safety and fatigue-related status in this group; as the primary analysis, to examine the extent to which sleep disturbance, sleep quality, and emotion regulation are associated with perceived workplace safety and fatigue-related status, operationalized with an author-developed data-collection tool; and to characterize the interrelations among the sleep and emotion-regulation measures themselves. Given the cross-sectional design and reliance on self-report, all findings are interpreted as associations; no temporal, causal, or intervention-related inferences are drawn.

## 2. Methods

### 2.1. Study design and participants

This study used a cross-sectional, correlational design. Participants were active-duty male firefighters serving in urban fire brigades in the Marmara region of Türkiye, each with a documented history of at least one occupational accident sustained during fire-service duties. The total number of firefighters approached before consent, and the number formally assessed for eligibility, were not recorded. Of 175 eligible firefighters who provided informed consent, 70 did not complete the full survey battery (partial or incomplete responses, n = 34; missing 1 or more core measures, n = 27; withdrawal or other reasons, n = 9), yielding a final analytic sample of 105 participants with complete data (completion rate 60.0%), as summarized in the Strengthening the Reporting of Observational Studies in Epidemiology participant-flow diagram (Fig. 1). Data were collected between January 2024 and June 2024 at 12 urban fire stations, and the survey was administered electronically. Individual station identifiers were not retained in the analytic dataset, so clustering of responses within the 12 stations could not be quantified or modeled. The analytic dataset contains complete data on every variable for all 105 participants; a complete-case approach was used, and no values were imputed. Occupational-accident history was reported by participants at enrollment and verified against the participating brigades’ institutional incident records. The date, type and severity of the index accident were not recorded, so accident history functions as an eligibility criterion rather than as a graded exposure. Age, fire-service experience, and rank were recorded at enrollment before the psychometric battery was administered and were therefore retained in the study enrollment records for all 70 individuals who consented but did not complete it, permitting a comparison of completers and non-completers on these 3 characteristics. Because eligibility required a documented accident history, the sample is not representative of the general firefighter population; this sampling frame is considered throughout the interpretation of findings. All participants provided informed electronic consent prior to accessing the survey. The study was approved by the Social and Humanities Research Ethics Committee of Istanbul University-Cerrahpasa (Approval No. 2023/219; May 2, 2023) and was conducted in accordance with the Declaration of Helsinki.^[37]^

**Figure 1.**
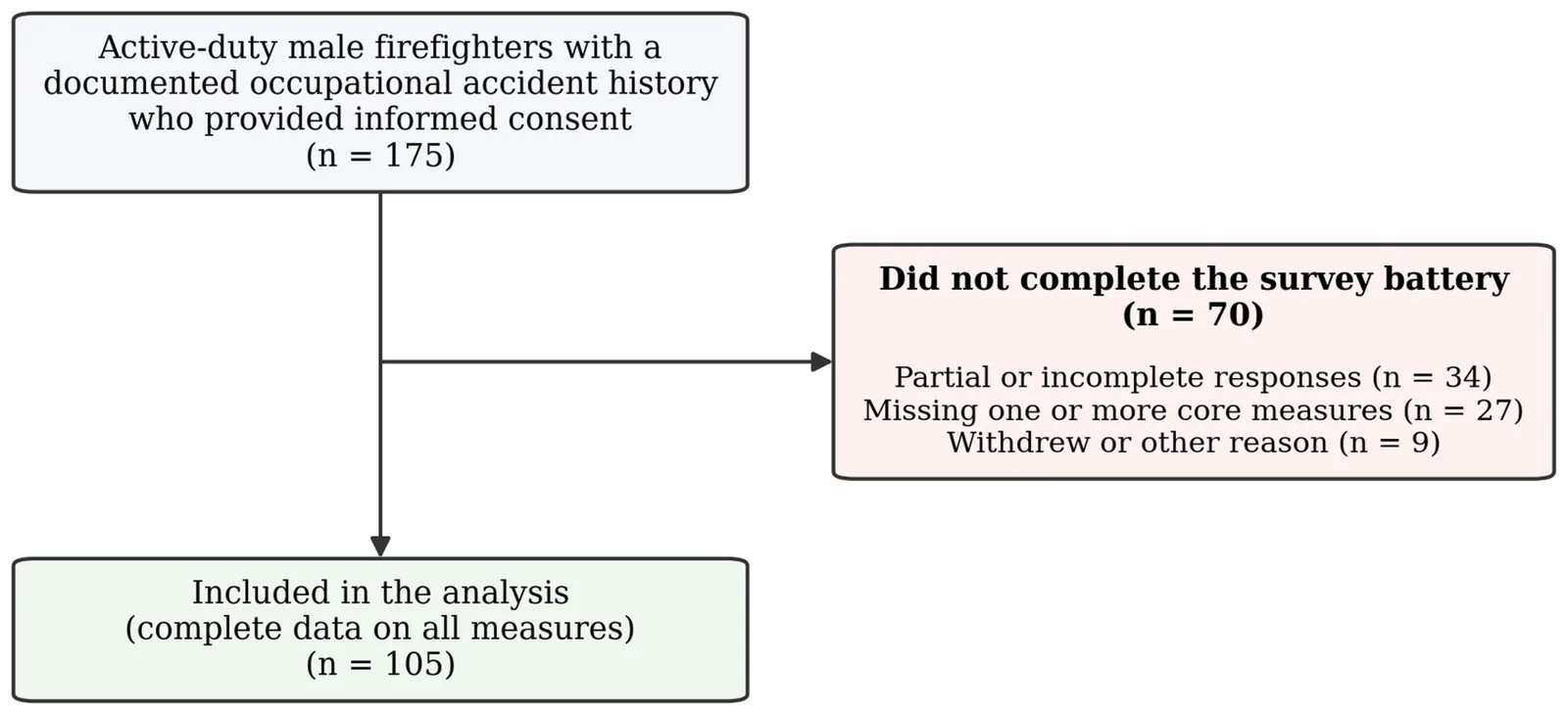
Participant flow (STROBE). Of 175 active-duty male firefighters with a documented occupational accident history who provided informed consent, 70 did not complete the survey battery (partial or incomplete responses, n = 34; 1 or more core measures missing, n = 27; withdrew or other reason, n = 9), leaving 105 participants with complete data. STROBE = Strengthening the Reporting of Observational Studies in Epidemiology.

### 2.2. Measures

#### 2.2.1. Sleep disturbance

Sleep disturbance was screened with the Basic Scale on Insomnia Complaints and Quality of Sleep (BaSIQS),^[38,39]^ a brief self-report measure comprising sleeping-problem and awakening-problem components (7 items, total score range 0 to 28); the Turkish version has shown acceptable reliability and validity. Item-level responses were not retained in the analytic dataset, so a sample-specific internal consistency coefficient could not be computed for this instrument; the 2 component scores correlated at *r* = .43.^[40]^ Higher total scores indicate greater sleep disturbance. Consistent with its screening purpose, BaSIQS scores are interpreted as reflecting the degree of sleep complaints rather than a clinical diagnosis of insomnia.

#### 2.2.2. Sleep quality

Global sleep quality was assessed with a single-item Sleep Quality Scale (SQS),^[41,42]^ on which higher ratings reflect better perceived sleep quality. The instrument presents a 0 to 10 discretizing visual analogue line grouped into 5 verbal categories (terrible, poor, fair, good, and excellent). In the present study, responses were recorded as those 5 categories and coded 0 (terrible) to 4 (excellent) rather than on the underlying 0 to 10 metric; observed values ranged from 1 to 4.

#### 2.2.3. Emotion regulation

Emotion-regulation skills were measured with the 27-item Emotion Regulation Skills Questionnaire (ERSQ),^[43,44]^ which assesses 9 skill domains (e.g., awareness, clarity, understanding, acceptance, tolerance, and modification of emotions). Higher scores indicate greater emotion-regulation skills. The total score ranges from 0 to 108. Internal consistency across the 9 subscale scores in the present sample was α = .96.

#### 2.2.4. Perceived workplace safety and fatigue

Perceived workplace safety and fatigue-related status was operationalized using the Perceived Workplace Safety/Fatigue Status Tool (PWSFS), a structured, author-developed data-collection tool (not a validated psychometric scale) that captures firefighters’ perceptions of workplace safety practices and fatigue-related status. The tool comprises 10 items rated on a 1 to 5 response format, organized into 4 content areas: safety awareness, fatigue management, health monitoring, and safety standards. Item responses were combined into a summary score by taking a weighted mean within each content area, multiplying by 20, and averaging the 4 area scores. Because the response format begins at 1, the resulting metric ranges from 20 to 100 rather than from 0 to 100; higher scores denote a more favorable perceived safety and fatigue status. The full item wording is provided in Table S1, Supplemental Digital Content 1 and the scoring formulas in Table S2, Supplemental Digital Content 2. The weights were assigned by the author on content grounds when the tool was constructed and were not derived empirically. The PWSFS is therefore best described as an exploratory, author-developed weighted composite intended to summarize selected perceived safety and fatigue-management indicators, and not as a validated psychometric scale: its 4 content areas are reported as prespecified content domains rather than as subscales, no interpretive cut-points are applied, and all PWSFS-based findings are treated as exploratory.

#### 2.2.5. Demographic and occupational characteristics

Participants reported age, years of fire-service experience (defined as total completed years of active fire-service duty, self-reported), rank, marital status, parental status, current smoking, and monthly income (expressed as multiples of the national minimum wage).

### 2.3. Sample size and statistical power

No a priori sample size calculation was performed. The achieved sample comprised 105 consenting participants who completed all core measures during the recruitment period, so sample size was determined by recruitment and completion rather than by a target precision. A sensitivity analysis was therefore conducted to establish the smallest effects the achieved sample could detect.^[45]^ With N = 105, 5 terms in the regression model, α = .05 (two-tailed), and power set at .80, the design was sensitive to an overall model effect of *f*^2^ = 0.13, corresponding to an *R*^2^ of approximately 0.11, and to a unique predictor contribution of *f*^2^ = 0.08, corresponding to a semipartial *R*^2^ of approximately 0.025 at the observed model *R*^2^. Effects smaller than these values may therefore have gone undetected. Observed (post hoc) power was not computed, because it is a monotonic function of the observed *P* value and adds no information beyond the confidence intervals (CIs) reported here. Sensitivity computations were based on the noncentral *F* distribution with noncentrality parameter λ = *f*^2^ × N.

### 2.4. Statistical analysis

Analyses were conducted in R (version 4.3.3; R Foundation for Statistical Computing) using the psych, car, lmtest, GPArotation, boot, sandwich, MASS, and readxl packages. A single annotated R script reproducing the analyses reported here is provided with this submission, together with the unedited console output of the run from which the reported values were taken. The de-identified analytical dataset is available from the author on reasonable request, subject to institutional and ethical restrictions. Continuous variables are summarized as means and standard deviations (SDs), and categorical variables as frequencies and percentages. Bivariate associations among sleep disturbance (BaSIQS), SQS, ERSQ, and perceived workplace safety and fatigue (PWSFS total) were examined with Pearson correlations, with Spearman correlations computed as a robustness check. The primary analysis was a multiple linear regression in which the PWSFS total score was the outcome, and sleep disturbance, sleep quality, and emotion regulation were entered as simultaneous predictors, adjusting for age and years of fire-service experience. Covariates in the primary model were specified a priori on conceptual grounds and deliberately kept few: with 105 observations and 5 terms, the model retains approximately 21 observations per term, which protects the precision of the coefficient of interest. The remaining recorded characteristics, namely current smoking, monthly income, marital status, parental status, and rank, were entered in an extended model reported as a sensitivity analysis rather than in the primary specification. Variables such as shift schedule, night-call frequency, workload, body mass index, chronic medical illness, medication use, and diagnosed mental health conditions were not recorded in this survey and could not be modeled; the resulting potential for residual confounding is addressed in the Limitations. For each predictor, we report unstandardized coefficients (*B*) with 95% CIs, standardized coefficients (β), and exact *P* values; overall model fit is reported as *R*^2^ and the associated *F* test, together with Cohen *f*^2^, computed as *R*^2^/(1 − *R*^2^).^[45]^ Regression assumptions were specified before analysis and examined as follows: normality of residuals with the Shapiro–Wilk test, homoscedasticity with the studentized Breusch–Pagan test, first-order residual autocorrelation in the data ordering with the Durbin–Watson statistic, multicollinearity with variance inflation factors, tolerances, and condition indices, linearity by inspection of residual-versus-fitted plots, and influential observations with Cook distance and leverage values. As a distribution-free check on the primary coefficient, a percentile bootstrap with 5000 resamples was also computed. All diagnostic results are reported in Section 3 and in Table S6, Supplemental Digital Content 3. Statistical significance was evaluated at α = .05 (two-tailed), and exact *P* values with 95% CIs are reported throughout. Because all constructs were assessed by self-report from a single source, the potential for common-method variance is addressed in Section 4. Given the cross-sectional design, all associations are interpreted as correlational; no causal or temporal inferences are drawn.

Several additional and sensitivity analyses were conducted. First, the measurement properties of the PWSFS were examined at the item level: Cronbach’s α for the full item set and for each content domain, corrected item-total correlations, α with each item deleted, and the Kaiser–Meyer–Olkin measure with Bartlett test of sphericity. Two separate procedures were then applied: principal-component parallel analysis against 1000 random data matrices to evaluate how many components the item set supports, and, separately, a forced 4-factor principal-axis factor analysis with varimax rotation to examine whether the 4 author-assigned content domains were empirically recovered. With 105 respondents and a Kaiser–Meyer–Olkin value near the lower acceptable bound, both are interpreted as exploratory only. These coefficients are reported descriptively rather than as criteria of adequacy, because the PWSFS was constructed as a weighted composite of deliberately distinct content domains and not as a set of interchangeable indicators of a single latent construct. Second, to test whether the association between sleep disturbance and perceived safety depends on shared item content, the primary analysis was repeated with 3 alternative outcomes: the composite excluding the fatigue-management domain; the safety-awareness domain alone, which comprises the only 3 items containing no reference to fatigue, sleep, or alertness; and the 7 items that do contain such references. Third, the predictors were entered hierarchically in 3 blocks, comprising the demographic covariates, then sleep quality and emotion regulation, then sleep disturbance, with Δ*R*^2^, its *F* test, and Cohen’s *f*^2^ reported for each block and partial and semipartial correlations for the full model. Fourth, an extended model added current smoking, monthly income, marital status, parental status, and rank to the primary specification; because of sparse cell sizes, rank was dichotomized as firefighter versus chief or sergeant. Fifth, 4 influence- and specification-sensitive analyses were run on the primary model: heteroscedasticity-consistent (HC3) standard errors, reestimation after deleting every observation flagged by conventional influence screening thresholds, an MM-estimator robust regression, and the leave-one-out range of the sleep-disturbance coefficient. Sixth, because the single-item sleep quality measure was recorded on a coarse ordinal metric, the primary model was reestimated with that variable entered as a set of indicator variables rather than as a continuous term. Seventh, because 2 items belonging to different content domains showed an unusually high level of response agreement, the composite was recomputed with each of those items removed, with both removed, and with all 10 items weighted equally, and each variant was carried through the primary regression. Finally, completers and non-completers were compared on the characteristics recorded at enrollment: age and fire-service experience with Welch *t* tests, reported with the mean difference, its 95% CI, and Hedges’ g, and rank with a chi-square test reported with Cramér’s V and corroborated by the Freeman–Halton exact test, because 1 expected cell count fell below 5. All analyses beyond the primary regression are exploratory, and their *P* values are reported without adjustment for multiplicity; they are interpreted as a pattern rather than through selective emphasis on individual coefficients.

## 3. Results

### 3.1. Descriptive statistics

Sample characteristics are presented in Table 1. On average, the BaSIQS mean was 12.2 (SD = 5.0; range 2–25) and the SQS mean was 2.4 (SD = 0.7), corresponding to a rating between fair and good on the 0 to 4 category coding, with an ERSQ mean of 74.1 (SD = 17.5). Perceived workplace safety and fatigue-related status averaged 73.2 (SD = 11.2; range 45.0–98.5) on the 20 to 100 metric; mean scores across the 4 content areas were 72.0 (safety awareness), 68.9 (fatigue management), 76.4 (health monitoring), and 75.4 (safety standards). No interpretive bands are applied to the composite because no validated cut-points exist for it.

**Table 1:** Sample characteristics.

Characteristic	Value (N = 105)
Age, yrs, M ± SD (range)	36.0 ± 6.9 (22–52)
Fire-service experience, yrs, M ± SD (range)	10.5 ± 6.3 (1–19)
Monthly income, minimum-wage multiples, M ± SD	3.5 ± 0.8 (2–6)
Rank, n (%)	Chief 4 (3.8); Sergeant 11 (10.5); Firefighter 90 (85.7)
Marital status, n (%)	Married 80 (76.2); Single 25 (23.8)
Has children, n (%)	Yes 68 (64.8); No 37 (35.2)
Current smoker, n (%)	Yes 52 (49.5); No 53 (50.5)

All participants were male active-duty firefighters with a documented occupational accident history.

SD = standard deviation.

Participants who completed the battery (n = 105) did not differ appreciably from those who consented but did not complete it (n = 70) on any characteristic recorded at enrollment (Table S7, Supplemental Digital Content 4). Mean age was 36.0 years (SD 6.9) among completers and 35.0 years (SD 7.5) among non-completers, a difference of 1.0 year (95% CI − 1.2 to 3.2; Welch *t*(139.4) = 0.88, *P* = .38; Hedges *g* = 0.14). Fire-service experience was 10.5 years (SD 6.3) and 9.7 years (SD 8.1), respectively, a difference of 0.8 years (95% CI − 1.4 to 3.1; Welch *t*(123.1) = 0.73, *P* = .46; Hedges g = 0.12). Rank distributions were also similar (chief 4 [3.8%] vs 6 [8.6%]; sergeant 11 [10.5%] vs 7 [10.0%]; firefighter 90 [85.7%] vs 57 [81.4%]; χ^2^(2) = 1.77, *P* = .41, Cramér V = 0.10; Freeman–Halton exact *P* = .41). No evidence of differential completion was therefore observed for the characteristics that could be examined; selection on unmeasured characteristics remains possible since motivation, current symptom burden, and workload at the time of data collection were not recorded.

### 3.2. Bivariate associations

Table 2 reports descriptive statistics and bivariate correlations. Greater sleep disturbance was strongly associated with less favorable perceived workplace safety and fatigue status (*r* = −.81, 95% CI [−.87, −.74], *P* < .001; Fig. 2), whereas better sleep quality (*r* = .52 [.37, .65], *P* < .001) and greater emotion-regulation skills (*r* = .44 [.27, .59], *P* < .001) were each associated with more favorable status. The sleep and emotion-regulation measures were themselves interrelated: sleep disturbance was associated with poorer sleep quality (*r* = −.60, *P* < .001) and lower emotion-regulation skills (*r* = −.58, *P* < .001), and better sleep quality was associated with greater emotion-regulation skills (*r* = .47, *P* < .001). Spearman coefficients were comparable in magnitude throughout.

**Table 2:** Descriptive statistics and bivariate correlations.

Variable	*M*	SD	1	2	3	4
1. Sleep disturbance (BaSIQS)	12.2	5.0	1.00			
2. Sleep quality (SQS)	2.4	0.7	−.60***	1.00		
3. Emotion regulation (ERSQ)	74.1	17.5	−.58***	.47***	1.00	
4. Perceived safety/fatigue (PWSFS)	73.2	11.2	−.81***	.52***	.44***	1.00

N = 105. Higher BaSIQS = greater sleep disturbance; higher SQS, ERSQ, and PWSFS = better/more favorable status. SQS was coded 0 (terrible) to 4 (excellent).

BaSIQS = Basic Scale on Insomnia Complaints and Quality of Sleep, ERSQ = Emotion Regulation Skills Questionnaire, PWSFS = Perceived Workplace Safety/Fatigue Status Tool, SD = standard deviation, SQS = Sleep Quality Scale.

*** *P* < .001.

**Figure 2.**
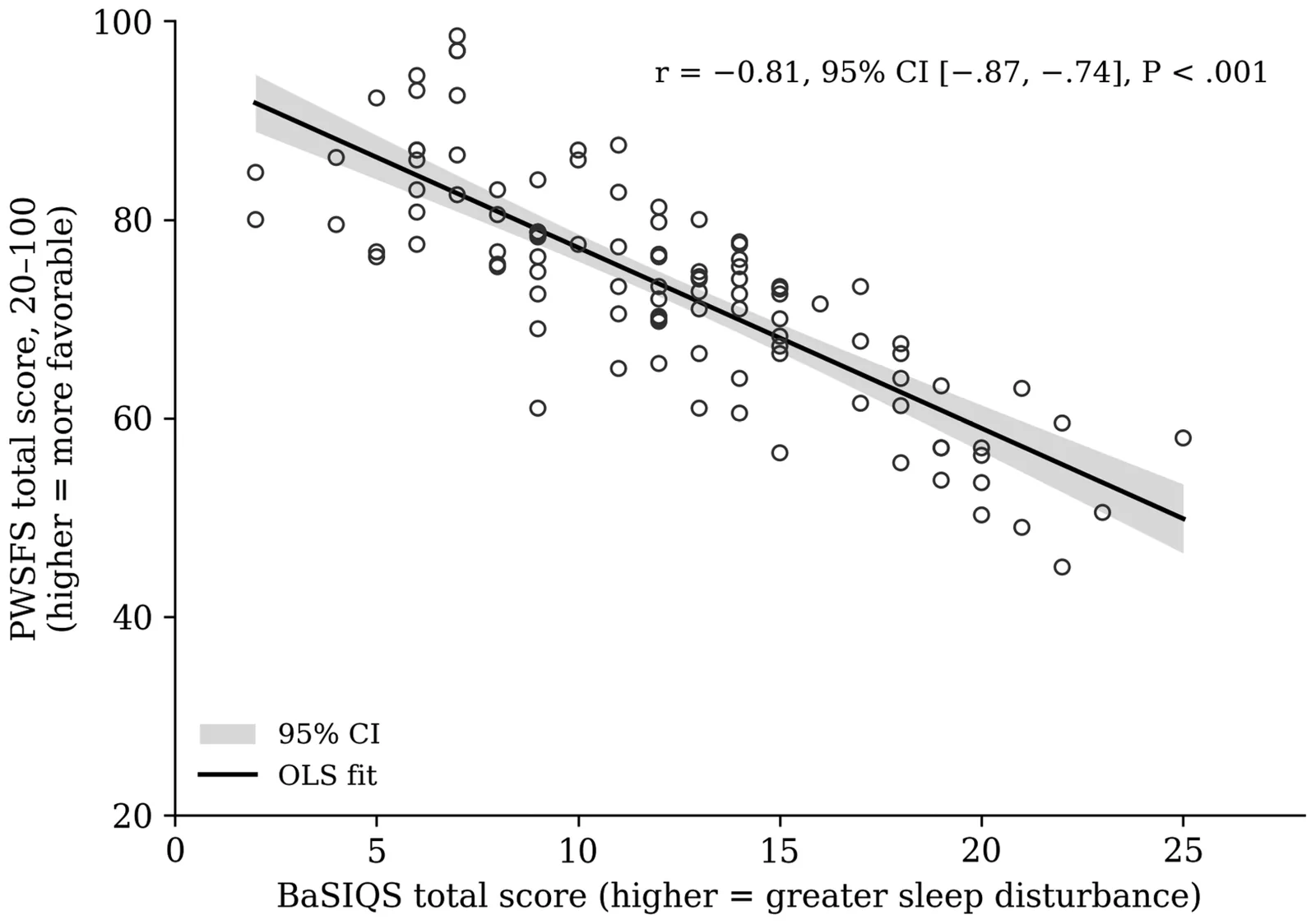
Association between sleep disturbance (BaSIQS) and perceived workplace safety and fatigue (PWSFS); N = 105. Circles represent individual participants, the solid line is the ordinary least squares fit, and the shaded band is the 95% confidence interval. Higher BaSIQS scores indicate greater sleep disturbance; higher PWSFS scores, on a 20 to 100 metric, indicate a more favorable perceived safety and fatigue status. BaSIQS = Basic Scale on Insomnia Complaints and Quality of Sleep, CI = confidence interval.

### 3.3. Correlates of perceived workplace safety and fatigue

In the multiple linear regression predicting perceived workplace safety and fatigue status (Table 3), the predictors together accounted for a substantial proportion of variance, *R*^2^ = .67, adjusted *R*^2^ = .65, F(5, 99) = 39.74, *P* < .001, Cohen *f*^2^ = 2.01. Sleep disturbance was the only unique correlate: each additional BaSIQS point was associated with a 1.81-point lower PWSFS score (*B* = −1.81, 95% CI [−2.17, −1.45], β = −.81, *P* < .001), accounting uniquely for 33.3% of the variance in the outcome (semipartial *r* = −.58, partial *r* = −.71, *f*^2^ = 1.00). After accounting for sleep disturbance, neither sleep quality (β = .06, *P* = .455) nor emotion regulation (β = −.05, *P* = .473) contributed uniquely, and age and fire-service experience were unrelated to the outcome (both *P* > .66). The bivariate associations of sleep quality and emotion regulation with perceived safety/fatigue thus appeared to be largely accounted for by their overlap with sleep disturbance. Regression diagnostics were satisfactory: residuals were approximately normal (Shapiro–Wilk *W* = .997, *P* = .999), there was no significant heteroscedasticity (studentized Breusch–Pagan LM = 4.97, df = 5, *P* = .42), there was no evidence of first-order residual autocorrelation in the data ordering (Durbin–Watson 1.90), although dependence among personnel within the same station could not be assessed because station identifiers were not retained. Multicollinearity was acceptable (variance inflation factors 1.6–3.1, tolerances .33–.63, maximum condition index 3.55), and no observation exceeded a Cook distance of 1 (maximum 0.09), although 9 observations exceeded the conventional 4/n screening threshold and 1 observation had high leverage (0.32), which prompted the influence-sensitive analyses reported below. A percentile bootstrap with 5000 resamples gave a 95% CI for the sleep-disturbance coefficient of − 2.18 to − 1.44, close to the parametric interval.

**Table 3:** Multiple linear regression predicting perceived workplace safety and fatigue (PWSFS).

Predictor	*B*	95% CI	β	*P*
Sleep disturbance (BaSIQS)	−1.81	[−2.17 to −1.45]	−.81	<.001
Sleep quality (SQS)	0.87	[−1.43 to 3.16]	.06	.455
Emotion regulation (ERSQ)	−0.03	[−0.13 to 0.06]	−.05	.473
Age	0.07	[−0.26 to 0.39]	.04	.692
Fire-service experience	−0.08	[−0.43 to 0.28]	−.04	.665

N = 105. *R*^2^ = .67, adjusted *R*^2^ = .65, *F*(5, 99) = 39.74, *P* < .001.

β = standardized coefficient, BaSIQS = Basic Scale on Insomnia Complaints and Quality of Sleep, CI = confidence interval, ERSQ = Emotion Regulation Skills Questionnaire, PWSFS = Perceived Workplace Safety/Fatigue Status Tool, SQS = Sleep Quality Scale.

### 3.4. Measurement properties of the PWSFS

Internal consistency for the full 10-item set was α = 0.71. Coefficients for the 4 content domains were lower: safety awareness 0.57, fatigue management 0.39, health monitoring 0.32 and safety standards 0.34. The last 2 corresponded to inter-item correlations of 0.19 and 0.20 and Spearman–Brown coefficients of 0.33 and 0.34. Corrected item-total correlations ranged from 0.11 to 0.55, and deleting any single item left α at or below 0.72 (Table 4). The item set was factorable (Kaiser–Meyer–Olkin 0.64; Bartlett χ^2^(45) = 256.8, *P* < .001). Principal-component parallel analysis supported 2 components rather than 4: the first 2 observed eigenvalues were 2.93 and 1.55, against corresponding 95th-percentile random eigenvalues of 1.66 and 1.46, and together accounted for 44.8% of the variance. A separate forced 4-factor principal-axis analysis with varimax rotation did not reproduce the 4 author-assigned content domains (Tables S3 and S4, Supplemental Digital Content 5): Q1, Q2, and Q8 loaded on a common factor; Q4 and Q5 loaded together; Q7 and Q9 loaded together; and Q3, Q6, and Q10 loaded on a fourth factor. Only the fatigue-management pair Q4 and Q5 partially reproduced an assigned domain, and Q4 also loaded on 2 other factors. Although Q1 and Q8 belong to different content domains, they correlated at *R* = 0.80 and carried identical responses in 82 of the 105 cases; this concordance is examined in the item-audit analyses reported below. All of these values are descriptive information about an exploratory composite rather than evidence for or against a 4-factor structure.

**Table 4:** PWSFS item statistics and internal consistency.

Item	*M*	SD	Corrected item-total *r*	α if item deleted
Q1 Awareness of safety measures	3.35	1.13	0.552	0.651
Q2 Awareness of long-term health risks	3.49	1.09	0.390	0.681
Q3 Open safety communication	4.04	1.06	0.358	0.686
Q4 Management of fatigue impact on teamwork	3.44	1.21	0.413	0.677
Q5 Attention under fatigue	3.65	1.07	0.316	0.694
Q6 Rest periods after operations	3.22	1.17	0.313	0.695
Q7 Health and fitness checks	4.19	0.97	0.237	0.705
Q8 Ergonomic conditions	3.45	1.07	0.532	0.656
Q9 Maintenance of safety standards	3.51	0.99	0.438	0.674
Q10 Regular safety updates	4.03	0.98	0.110	0.723

N = 105; items were rated from 1 to 5. Cronbach α was 0.71 for the full 10-item set, 0.57 for safety awareness (Q1–Q3), 0.39 for fatigue management (Q4–Q6), 0.32 for health monitoring (Q7–Q8), and 0.34 for safety standards (Q9–Q10). These coefficients are descriptive because the PWSFS is an author-developed weighted composite of heterogeneous indicators rather than a reflective psychometric scale.

PWSFS = Perceived Workplace Safety/Fatigue Status Tool, SD = standard deviation.

### 3.5. Hierarchical regression and unique contributions

Entered hierarchically, age and fire-service experience together accounted for 2.3% of the variance in perceived workplace safety and fatigue and did not reach significance (*F*(2, 102) = 1.22, *P* = .30). Adding sleep quality and emotion regulation raised *R*^2^ to .335 (Δ*R*^2^ = .311, *F*(2, 100) = 23.40, *P* < .001, *f*^2^ = 0.47). Adding sleep disturbance raised it to .667 (Δ*R*^2^ = .333, *F*(1, 99) = 99.06, *P* < .001, *f*^2^ = 1.00). Sleep disturbance therefore explained an additional 33.3% of the variance when entered after the preceding predictors, and in the full model the remaining 4 predictors together contributed <0.5% uniquely (Table 5). Because incremental contributions in hierarchical models depend on entry order, these values describe the chosen sequence rather than a fixed partition of variance.

**Table 5:** Hierarchical regression predicting perceived workplace safety and fatigue, and unique contributions in the full model.

Panel A. Hierarchical blocks	*R* ^2^	ΔR^2^	F for ΔR^2^	*P*	*f* ^2^
1. Age, fire-service experience	.023	.023	*F*(2, 102) = 1.22	.299	0.02
2. + sleep quality, emotion regulation	.335	.311	*F*(2, 100) = 23.40	<.001	0.47
3. + sleep disturbance	.667	.333	*F*(1, 99) = 99.06	<.001	1.00
Panel B. Unique contributions	sr	sr^2^	Partial *r*	*P*	*f* ^2^
Sleep disturbance	−.58	.333	−.71	<.001	1.00
Sleep quality	.04	.002	.08	.455	0.01
Emotion regulation	−.04	.002	−.07	.473	0.01
Age	.02	.001	.04	.692	0.00
Fire-service experience	−.03	.001	−.04	.665	0.00

N = 105. Panel A gives block-level statistics for the hierarchical sequence; Panel B gives the unique contribution of each predictor in the full model. Incremental contributions in Panel A depend on entry order.

sr = semipartial correlation.

### 3.6. Sensitivity analyses

Because the PWSFS contains items referring to fatigue, sleep, and alertness, the primary analysis was repeated with 3 alternative outcomes (Table 6). Removing the fatigue-management area reduced the zero-order correlation from − .81 to − .75 and the model *R*^2^ from .667 to .570, while the standardized coefficient for sleep disturbance moved from − .81 to − .79. Restricting the outcome to the 3 safety-awareness items, the only items containing no reference to fatigue, sleep, or alertness, reduced the correlation further to − .58 and the model *R*^2^ to .384, with a standardized coefficient of − .77. The association therefore persisted in every reduced-content variant but was materially attenuated: relative to the full composite, the absolute correlation fell by approximately 29% and the explained variance by approximately 42%. Restricting the outcome to the 7 items that do refer to fatigue, sleep, or alertness gave a correlation of − .80 and a standardized coefficient of − .71. At the item level, the 2 items most strongly associated with sleep disturbance were Q9 (*r* = −.58), which refers to alertness, and Q1 (*r* = −.53), which does not, while the weakest association was for Q10 (*r* = −.20), which refers to sleep health.

**Table 6:** Sensitivity of the sleep-disturbance association to the composition of the PWSFS outcome.

Outcome	M ± SD	α	*r* with BaSIQS [95% CI]	*R* ^2^	β for BaSIQS
Full PWSFS (4 content domains)	73.2 ± 11.2	0.71	−.81 [−.87 to −.74]	.667	−.81
Excluding fatigue management	74.6 ± 11.6	0.64	−.75 [−.82 to −.65]	.570	−.79
Safety awareness only (Q1–Q3)	72.0 ± 16.2	0.57	−.58 [−.69 to −.44]	.384	−.77
Fatigue, sleep or alertness items only (Q4–Q10)	72.8 ± 11.4	0.59	−.80 [−.86 to −.72]	.656	−.71

N = 105. Model specification identical in every row: sleep disturbance, sleep quality, and emotion regulation, adjusted for age and fire-service experience. The safety-awareness variant contains the only 3 items with no reference to fatigue, sleep, or alertness. Correlations are reported as observed; no correction for attenuation is applied because Cronbach α is not an appropriate reliability estimate for a weighted composite of deliberately heterogeneous content.

BaSIQS = Basic Scale on Insomnia Complaints and Quality of Sleep, CI = confidence interval, PWSFS = Perceived Workplace Safety/Fatigue Status tool, SD = standard deviation.

In an extended model adding current smoking, monthly income, marital status, parental status, and rank to the primary specification, the coefficient for sleep disturbance was essentially unchanged (*B* = −1.83, 95% CI − 2.23 to − 1.44, β = −.82, *P* < .001; model *R*^2^ = .687, adjusted *R*^2^ = .654). None of the added covariates was associated with the outcome except marital status (*B* = 4.46, *P* = .042), and its inclusion did not alter the interpretation of any other term (Table S5, Supplemental Digital Content 6).

The primary coefficient was stable across influence- and specification-sensitive analyses. With HC3 standard errors, the point estimate for sleep disturbance was identical (*B* = −1.81, robust SE 0.20 against 0.18, *t* = −9.05, *P* < .001). Nine observations were flagged by either the 4/n Cook distance threshold or the leverage criterion, the single high-leverage case being among them; reestimation after excluding those 9 gave *B* = −1.92 with *R*^2^ = .731 in the remaining 96 cases. An MM-estimator robust regression gave *B* = −1.83, and across all 105 leave-one-out refits, the coefficient ranged from − 1.89 to − 1.74. Entering sleep quality as a set of indicator variables rather than as a continuous term gave *B* = −1.76 for sleep disturbance with *R*^2^ = .695, and no individual sleep-quality level was associated with the outcome (Table S8, Supplemental Digital Content 7).

### 3.7. Item audit and weighting sensitivity

Because Q1 and Q8 belong to different content domains yet carried identical responses in 82 of the 105 cases, the raw item export was audited. Within the available item export, the 2 columns are distinct: they differ in 23 cases, with differences ranging from − 3 to + 2, so no evidence of direct column duplication was identified, and the full cross-tabulation is given in Table S9, Supplemental Digital Content 8. The export was not additionally remapped against the original paper form, so upstream label assignment cannot be verified beyond the exported variable names. Uniform responding does not explain the pattern either, since no respondent gave the same answer to all 10 items, and every respondent used between 2 and 5 of the 5 available response options. The concordance is also specific to this pair rather than general to the instrument, the next highest agreement between any 2 items being 49.5% for Q4 and Q5. Within the limits of that check, the pattern appears to be a property of these 2 items rather than a data-handling artifact, and may reflect content redundancy or response assimilation, in that an appraisal of general safety-measure awareness and an appraisal of ergonomic provision may have been answered as a single judgment by most respondents. Recomputing the composite without either item, without both, and with all 10 items weighted equally left the association with sleep disturbance essentially intact (Table 7). The unweighted composite correlated with the published weighted composite at *r* = .99, indicating that the author-assigned weights make little practical difference to the results.

**Table 7:** Sensitivity of the sleep-disturbance association to individual items and to the weighting scheme.

Outcome variant	*M* ± SD	α	*r* with BaSIQS [95% CI]	*R* ^2^	β for BaSIQS
Full PWSFS, published weights	73.2 ± 11.2	0.71	−.81 [−.87 to −.74]	.667	−.81
All 10 items weighted equally	72.7 ± 11.3	0.71	−.81 [−.87 to −.73]	.660	−.82
Excluding Q1	74.0 ± 10.9	0.65	−.80 [−.86 to −.71]	.641	−.79
Excluding Q8	75.0 ± 10.9	0.66	−.79 [−.85 to −.71]	.638	−.73
Excluding both Q1 and Q8	75.8 ± 10.9	0.61	−.75 [−.83 to −.66]	.580	−.68

N = 105. Model specification identical in every row. The published weighted composite and the unweighted composite correlate at *r* = .99.

BaSIQS = Basic Scale on Insomnia Complaints and Quality of Sleep, CI = confidence interval, PWSFS = Perceived Workplace Safety/Fatigue Status tool, SD = standard deviation.

## 4. Discussion

This cross-sectional study examined how sleep disturbance, sleep quality, and emotion-regulation capacity relate to perceived workplace safety and fatigue-related status among active-duty male firefighters with a documented history of occupational accidents. Perceived safety and fatigue status was most strongly associated with sleep disturbance: firefighters reporting greater sleep complaints perceived their workplace as less safe and themselves as more fatigue-burdened, and sleep disturbance remained the only unique correlate in a multivariable model that also included sleep quality, emotion regulation, age, and years of fire-service experience. Better sleep quality and greater emotion-regulation skills were each associated with more favorable perceptions at the bivariate level but added no variance once sleep disturbance was considered. Because the design is cross-sectional and all constructs were self-reported, these findings are interpreted as associations rather than as evidence of causal or temporal relations.

The prominence of sleep disturbance is consistent with a growing literature linking impaired sleep and accumulated fatigue to safety-relevant outcomes in the fire service. Disturbed sleep and fatigue have been associated with diminished alertness and occupational performance,^[25,26]^ and firefighter fatigue has been linked to safety behavior, with perceived safety climate playing a moderating and mediating role.^[46]^ Sleep problems are highly prevalent in this workforce^[16]^ and appear difficult to resolve even away from duty,^[17]^ plausibly shaping how safe and capable firefighters feel at work. The present results extend this work by showing that firefighters’ own appraisal of workplace safety and fatigue tracks their sleep complaints closely, which places perceived safety alongside the more frequently studied behavioral and performance outcomes.

The magnitude of this association can be expressed in the units of the measures themselves. A one-point higher BaSIQS score corresponded to a 1.81-point lower PWSFS score, so a difference of 1 SD in sleep complaints (5.0 BaSIQS points) corresponded to a difference of 9.1 PWSFS points, or 0.81 SDs of the outcome. No further extrapolation is offered. Differences implied across the full observed range of the predictor would require the linear model to hold throughout a bounded outcome, no minimal important difference has been established for this composite, and no validated cut-points exist against which a change could be called operationally meaningful. The translation is therefore an aid to reading the scale units of a between-person association, not a statement about what a change in sleep would produce.

That sleep quality and emotion regulation showed bivariate but not unique associations likely reflects their substantial intercorrelation with sleep disturbance (rs = −.60 and − .58): firefighters reporting fewer sleep complaints also tended to report better sleep quality and greater regulatory skill. This pattern is consistent with evidence that sleep and emotion regulation are closely interrelated in high-stress responders,^[33–35]^ suggesting that, in this sample, shared variance among these constructs, rather than emotion regulation on its own, was associated with perceived safety and fatigue. This does not imply that emotion regulation is unimportant; its association with perceived safety may operate through, or overlap with, sleep-related experience.

Several features of the design temper interpretation of the strong sleep–safety association. First, the PWSFS overlaps with the sleep measures at the level of item content. The fatigue-management content area contributes 25% of the total score by construction, and 7 of the 10 items refer explicitly to fatigue, sleep, or alertness, the 3 safety-awareness items being the only ones that do not (Table S1, Supplemental Digital Content 1). The sensitivity analyses show that the association survives removal of the overlapping content but is weakened by it. The standardized coefficient moved only from − .81 to − .77, yet the zero-order correlation fell from − .81 to − .58 and the explained variance from 67% to 38% when only the 3 items with no fatigue, sleep, or alertness wording were retained. Shared item content therefore contributes to the observed association without fully explaining it, and the magnitude reported for the full composite should be read as an upper bound. Second, because sleep, emotion regulation, and perceived safety were all assessed by self-report from a single source at a single time point, common-method variance may have inflated the observed associations.^[47]^ The strength of the sleep–safety association should therefore be interpreted cautiously, as partly reflecting shared method and content rather than a precise estimate of a substantive relationship, and it provides no basis for causal claims, such as that improving sleep would raise perceived safety.^[48]^

The measurement properties of the PWSFS are weak by the standards applied to validated scales. Internal consistency for the full item set was 0.71, the 4 content domains fell between 0.32 and 0.57, and parallel analysis retained 2 components rather than 4. The instrument was assembled to cover distinct aspects of perceived safety and fatigue with weights assigned on content grounds, so it is a composite index rather than a reflective scale, and internal homogeneity was never a design objective. That reading is offered as a description of how the tool was built and not as a defense of the coefficients: the evidence normally required of an index, including a documented item-generation process, expert content review, cognitive pretesting, an empirical rationale for the weights, and external criterion validation, has not been assembled for this instrument. The practical consequences are that the 4 domains cannot be read as subscales, that the total score should be treated as an exploratory summary rather than a measurement of a defined construct, and that every finding involving it requires replication with a validated safety-climate instrument.

The direction of these associations cannot be established from these data, and more than one account fits them equally well. Sleep complaints may shape how safe and fatigue-resilient firefighters judge their working environment to be. The reverse ordering is at least as plausible: personnel who regard their station as poorly resourced, their rest periods as inadequately protected, or their safety standards as loosely maintained may sleep worse for those reasons. A third possibility is that both appraisals are downstream of factors not measured here, among them organizational climate, workload during the preceding period, unresolved consequences of the index accident, and general psychological distress, any of which could produce the observed correlation without a direct link between sleep and perceived safety. A single-occasion, single-source design does not distinguish these accounts, and the present results should not be read as indicating that improving sleep would raise perceived workplace safety.

The findings nonetheless point to a tentative but practically relevant pattern: among firefighters with prior occupational accidents, sleep complaints are closely tied to how safe and fatigue-resilient they perceive themselves to be. If corroborated in longitudinal, multi-source designs, this would support attention to sleep health within firefighter safety and fatigue-management programs,^[26,27]^ alongside organizational efforts to strengthen safety climate.^[14]^ Because perceptions of safety can themselves shape safety behavior,^[12,14,46]^ understanding their individual-level correlates may complement organizational approaches.

## 5. Strengths and limitations

This study has several strengths: it focused on an under-examined but practically important outcome, firefighters’ perceptions of workplace safety and fatigue, in a specific, policy-relevant group (personnel with documented occupational accidents); it used the validated BaSIQS and ERSQ instruments together with a modified categorical administration of the single-item SQS; and it reported associations with exact *P* values, CIs, and regression diagnostics. Several limitations must be weighed. First, the cross-sectional design precludes causal or temporal inference; the observed associations are equally compatible with reverse or reciprocal relations. Second, participants were a self-selected sample of male firefighters with a documented accident history, and approximately 40% of those who consented did not complete the battery. No evidence of differential completion was observed for age, fire-service experience, or rank (Table S7, Supplemental Digital Content 4). Selection on unmeasured characteristics nonetheless remains possible because motivation, current symptom burden, and workload at the time of data collection were not recorded, and the findings should not be extrapolated to firefighters without accident histories or to the workforce at large. The sample contained no women; the available records did not permit determination of whether this reflected the composition of the participating brigades or differential participation, and the findings cannot be extended to female firefighters. Third, all constructs were measured by self-report at a single time point, raising the possibility of common-method variance. In addition, sleep quality was recorded using the 5 verbal categories of the single-item scale and coded 0 to 4 rather than on the validated 0 to 10 metric, which discards information and would be expected to attenuate any association involving that variable; the absence of a unique contribution from sleep quality in the multivariable model should be read in that light. Fourth, the PWSFS is an author-developed data-collection tool rather than a validated psychometric scale; its content domains were treated as item groups, its internal consistency was modest (α = 0.71 for the full item set and 0.32–0.57 within content domains), and exploratory factor analysis did not recover the 4-domain structure. The composite should therefore be regarded as an exploratory index rather than a measure with established structural validity, although the sensitivity analyses indicate that its overlap with fatigue-related content contributes to, but does not fully explain, the association reported here. A single 5-point response anchor was applied to all 10 items, and that anchor sits more naturally with the items asking about effectiveness or frequency than with those asking about awareness, being informed, or freedom to communicate; this semantic mismatch is a further measurement limitation of the instrument. Fifth, the primary model adjusted only for age and fire-service experience. Current smoking, rank, income, marital status, and parental status were additionally modeled in a sensitivity analysis, which left the coefficient for sleep disturbance unchanged, but shift schedule, night-call frequency, workload, body mass index, chronic medical illness, medication use, and diagnosed mental health conditions were not measured at all. Several of these plausibly influence both sleep complaints and perceptions of workplace safety, and if unmeasured and associated with both in the same direction, they would inflate the observed association; residual confounding therefore cannot be excluded. Sixth, firefighters are exposed to several occupational hazards at the same time, among them combustion products, heat, physical load, noise, and shift work, and none of these was measured in this survey. Where exposures co-occur in this way, the contribution of any single one cannot be isolated, so sleep disturbance should be read here as one correlate within a hazard profile rather than as an exposure that can be separated from it. Participants were also drawn from 12 fire stations, several outcome items refer to station-level conditions and organizational practice, and responses from personnel in the same station may therefore be correlated; station identifiers were not retained in the analytic dataset, so this clustering could not be quantified or modeled. With approximately 9 participants per station, the design is one in which intraclass correlation could meaningfully affect precision, and the standard errors reported here should be regarded as possibly optimistic. Finally, the accident history was a sampling characteristic rather than a measured exposure, and its recency, severity, and circumstances were not characterized.

Several design features would strengthen the next study in this line. Prospective, repeated-measures designs would allow sleep and safety appraisals to be ordered in time rather than observed together. Objective sleep measurement by actigraphy or polysomnography would remove the method shared by the sleep and safety measures here, and a validated organizational safety climate instrument would separate the individual appraisal from the organizational property it partly reflects. Linking these measures to administratively recorded outcomes, including subsequent accidents, near-miss reports, and injury claims, would test whether perceived safety tracks anything beyond itself. Recording the co-exposures characteristic of the profession, among them combustion-product and heat exposure, call volume, and shift pattern, would allow the contribution of sleep to be estimated alongside the other hazards rather than in isolation.

Overall, these findings position perceived workplace safety and fatigue as an individual-level construct worth studying in its own right and identify sleep as its most salient correlate in this sample, while leaving the strength of that link, and its direction, as open questions. Bridging the individual-difference and organizational traditions in future longitudinal, multi-source research should determine whether sleep-related interventions alter perceived safety and, separately, validated behavioral or incident-based safety outcomes.

## 6. Conclusions

Among active-duty male firefighters with a documented history of occupational accidents, sleep disturbance was, among the individual-level variables measured in this study, the factor most strongly associated with perceived workplace safety and fatigue-related status: firefighters reporting more sleep complaints perceived their environment as less safe and themselves as more fatigued. Sleep quality and emotion-regulation skills were associated with these perceptions at the bivariate level but added no unique variance beyond sleep disturbance. Because the study was cross-sectional and relied on single-source self-report, and because the perceived-safety tool is an exploratory composite whose 4 content areas were not recovered as separate dimensions, these associations are provisional and cannot support causal claims. The association persisted after removal of the most directly overlapping sleep- and fatigue-related items, although its magnitude was materially attenuated. These findings should be read as hypothesis-generating: the outcome composite has not undergone external validation, all variables were self-reported on a single occasion, and the cross-sectional design precludes temporal or causal inference. Sleep disturbance may be a relevant marker to consider within broader fatigue-risk and occupational-safety assessment, but whether addressing it changes safety outcomes requires prospective or experimental evaluation. Longitudinal studies incorporating objective sleep indicators and validated behavioral or incident-based safety outcomes are required.

## Acknowledgments

No individuals other than the author contributed to the study in a capacity warranting acknowledgments.

## Author contributions

**Conceptualization:** Ahmet Erhan Bakirci.

**Data curation:** Ahmet Erhan Bakirci.

**Formal analysis:** Ahmet Erhan Bakirci.

**Investigation:** Ahmet Erhan Bakirci.

**Methodology:** Ahmet Erhan Bakirci.

**Validation:** Ahmet Erhan Bakirci.

**Visualization:** Ahmet Erhan Bakirci.

**Writing – original draft:** Ahmet Erhan Bakirci.

**Writing – review & editing:** Ahmet Erhan Bakirci.


















