# Supplementary material for: Associations of sleep disturbance and emotion regulation with perceived workplace safety and fatigue among male firefighters with prior occupational accidents: A cross-sectional study
Source: Medicine (Baltimore). 2026 Oct 23;105(43):e51154. doi: 10.1097/MD.0000000000051154 (PMC13706495; doi:10.1097/MD.0000000000051154)
Supplement: Supplementary file 1 [file medi-105-e51154-s001.docx]

*Associations of Sleep Disturbance and Emotion Regulation With Perceived Workplace Safety and Fatigue Among Male Firefighters With Prior Occupational Accidents: A Cross-Sectional Study*

**Supplementary Table S1.** *Perceived Workplace Safety/Fatigue Status (PWSFS) tool: item content and content-area weighting.*

| **Item** | **English wording** | **Turkish wording (as administered)** | **Content domain** | **Weight within domain** |
| --- | --- | --- | --- | --- |
| Q1 | How would you rate your awareness of workplace safety measures in your current position? | Mevcut görevinizde iş yeri güvenlik önlemlerine ilişkin farkındalığınızı nasıl değerlendirirsiniz? | SAS | 40% |
| Q2 | To what extent are you informed about the long-term health risks associated with your job? | Mesleğinizle ilişkili uzun vadeli sağlık riskleri konusunda ne ölçüde bilgilendirilmiş durumdasınız? | SAS | 30% |
| Q3 | How freely can you communicate your safety concerns in your workplace? | İş yerinizde güvenlikle ilgili kaygılarınızı ne ölçüde rahatça dile getirebiliyorsunuz? | SAS | 30% |
| Q4 | How effectively is the effect of fatigue on team performance managed in your workplace? | İş yerinizde yorgunluğun ekip performansı üzerindeki etkisi ne ölçüde etkili biçimde yönetiliyor? | FMS | 35% |
| Q5 | How well do you maintain attention to safety regulations during high-fatigue periods? | Yorgunluğun yoğun olduğu dönemlerde güvenlik kurallarına dikkatinizi ne ölçüde koruyabiliyorsunuz? | FMS | 35% |
| Q6 | How effectively are rest periods managed after intensive operations? | Yoğun operasyonların ardından dinlenme süreleri ne ölçüde etkili biçimde yönetiliyor? | FMS | 30% |
| Q7 | How regularly are health and fitness checks conducted with consideration for sleep health? | Uyku sağlığı da gözetilerek sağlık ve kondisyon kontrolleri ne sıklıkta düzenli olarak yapılıyor? | HMS | 50% |
| Q8 | How well are ergonomic conditions established to reduce fatigue-related risks? | Yorgunluğa bağlı riskleri azaltmak üzere ergonomik koşullar ne ölçüde iyi biçimde sağlanmış durumda? | HMS | 50% |
| Q9 | How effectively are high safety standards maintained considering staff alertness levels? | Personelin uyanıklık düzeyi gözetilerek yüksek güvenlik standartları ne ölçüde etkili biçimde sürdürülüyor? | SSS | 50% |
| Q10 | How frequently are safety measures updated to account for sleep health concerns? | Uyku sağlığına ilişkin kaygıları dikkate alacak biçimde güvenlik önlemleri ne sıklıkta güncelleniyor? | SSS | 50% |

*Note. PWSFS = Perceived Workplace Safety/Fatigue Status tool; SAS = safety awareness; FMS = fatigue management; HMS = health monitoring; SSS = safety standards.* The instrument was administered in Turkish; both the administered Turkish wording and its English rendering are given above. Items are rated on a five-point response scale (1 = not at all effective/never to 5 = extremely effective/always; Turkish: 1 = hiç etkili değil / hiçbir zaman, 5 = son derece etkili / her zaman). *The four content areas contribute equally (25% each) to the PWSFS total score. The PWSFS is an author-developed data-collection tool rather than a validated psychometric scale; the content areas are treated as item groups and the weighting scheme is descriptive.* Seven of the ten items (Q4 to Q10) refer explicitly to fatigue, sleep, or alertness; the three safety-awareness items (Q1 to Q3) do not. This content overlap with the sleep measures is taken into account in the interpretation of the association between the PWSFS and the BaSIQS. The English rendering of Q4 shown here was corrected at revision. The previously submitted version contained a transcription error that phrased the item as an impact question rather than an effectiveness question, which was inconsistent with the shared response anchor and with the scoring direction of the composite. The Turkish wording, the item coding and the data are unchanged, and no reported result is affected.
