# Supplementary material for: Associations of sleep disturbance and emotion regulation with perceived workplace safety and fatigue among male firefighters with prior occupational accidents: A cross-sectional study
Source: Medicine (Baltimore). 2026 Oct 23;105(43):e51154. doi: 10.1097/MD.0000000000051154 (PMC13706495; doi:10.1097/MD.0000000000051154)
Supplement: Supplementary file 2 [file medi-105-e51154-s002.docx]

*Associations of Sleep Disturbance and Emotion Regulation With Perceived Workplace Safety and Fatigue Among Male Firefighters With Prior Occupational Accidents: A Cross-Sectional Study*

**Supplementary Table S2.** *PWSFS component scoring methodology.*

Each content-area score is computed as a weighted average of its item responses and multiplied by 20. Because the response format begins at 1, the resulting metric ranges from 20 to 100 rather than from 0 to 100.

| **Content area** | **Scoring formula** |
| --- | --- |
| Safety awareness (SAS) | (Q1 × 0.40 + Q2 × 0.30 + Q3 × 0.30) × 20 |
| Fatigue management (FMS) | (Q4 × 0.35 + Q5 × 0.35 + Q6 × 0.30) × 20 |
| Health monitoring (HMS) | (Q7 × 0.50 + Q8 × 0.50) × 20 |
| Safety standards (SSS) | (Q9 × 0.50 + Q10 × 0.50) × 20 |
| PWSFS total | (SAS + FMS + HMS + SSS) / 4 |

*Note. Higher PWSFS scores denote a more favorable perceived safety/fatigue status.* The interpretive bands reported in the previously submitted version have been withdrawn: they were defined on the assumption of a 0–100 metric, and no validated cut-points exist for this composite.
