# Supplementary material for: Associations of sleep disturbance and emotion regulation with perceived workplace safety and fatigue among male firefighters with prior occupational accidents: A cross-sectional study
Source: Medicine (Baltimore). 2026 Oct 23;105(43):e51154. doi: 10.1097/MD.0000000000051154 (PMC13706495; doi:10.1097/MD.0000000000051154)
Supplement: Supplementary file 3 [file medi-105-e51154-s003.docx]

*Associations of Sleep Disturbance and Emotion Regulation With Perceived Workplace Safety and Fatigue Among Male Firefighters With Prior Occupational Accidents: A Cross-Sectional Study*

**Supplementary Table S6. Regression assumption diagnostics for the primary model.**

| **Assumption** | **Test** | **Result** |
| --- | --- | --- |
| Normality of residuals | Shapiro-Wilk | W = 0.997, P = .999 |
| Homoscedasticity | Studentized Breusch-Pagan | LM = 4.97, df = 5, P = .420 |
| Homoscedasticity | Score test on fitted values | χ²(1) = 2.94, P = .086 |
| First-order residual autocorrelation in the data ordering | Durbin-Watson | 1.901 |
| Multicollinearity | Variance inflation factors | 1.58 to 3.06 (tolerance 0.33 to 0.63) |
| Multicollinearity | Maximum condition index | 3.55 |
| Influential observations | Cook's distance | maximum 0.093; none above 1; 9 above 4/n |
| Leverage | Hat values | maximum 0.320; 1 above 3p/n |
| Coefficient stability | Percentile bootstrap, 5000 resamples | B = −1.809, 95% CI [−2.179, −1.440] |
| Note. N = 105. Primary model: PWSFS total regressed on sleep disturbance, sleep quality, emotion regulation, age and fire-service experience. The parametric interval for the sleep-disturbance coefficient was [−2.170, −1.449]. | | |
