# Supplementary material for: Associations of sleep disturbance and emotion regulation with perceived workplace safety and fatigue among male firefighters with prior occupational accidents: A cross-sectional study
Source: Medicine (Baltimore). 2026 Oct 23;105(43):e51154. doi: 10.1097/MD.0000000000051154 (PMC13706495; doi:10.1097/MD.0000000000051154)
Supplement: Supplementary file 4 [file medi-105-e51154-s004.docx]

*Associations of Sleep Disturbance and Emotion Regulation With Perceived Workplace Safety and Fatigue Among Male Firefighters With Prior Occupational Accidents: A Cross-Sectional Study*

**Supplementary Table S7. Comparison of participants who completed the survey battery with those who did not.**

| **Characteristic** | **Completers (n = 105)** | **Non-completers (n = 70)** | **Difference [95% CI]** | **Test** | **Effect size** |
| --- | --- | --- | --- | --- | --- |
| Age, years, M ± SD (range) | 36.0 ± 6.9 (22–52) | 35.0 ± 7.5 (24–54) | 1.0 [−1.2, 3.2] | t(139.4) = 0.88, P = .38 | g = 0.14 |
| Fire-service experience, years, M ± SD (range) | 10.5 ± 6.3 (1–19) | 9.7 ± 8.1 (2–22) | 0.8 [−1.4, 3.1] | t(123.1) = 0.73, P = .46 | g = 0.12 |
| Rank, n (%) |  |  |  | χ²(2) = 1.77, P = .41 | V = 0.10 |
| Chief | 4 (3.8) | 6 (8.6) |  |  |  |
| Sergeant | 11 (10.5) | 7 (10.0) |  |  |  |
| Firefighter | 90 (85.7) | 57 (81.4) |  |  |  |
| Note. Characteristics were recorded at enrolment, before the psychometric battery was administered, and were therefore available for all 175 individuals who provided consent. Continuous variables were compared with Welch t tests because the variances were unequal for fire-service experience (variance ratio F(69, 104) = 1.64, P = .022). Rank was compared with a chi-square test; one expected cell count fell below five, and the Freeman-Halton exact test gave P = .41. g = Hedges g; V = Cramér V. | | | | | |
