# Supplementary material for: Associations of sleep disturbance and emotion regulation with perceived workplace safety and fatigue among male firefighters with prior occupational accidents: A cross-sectional study
Source: Medicine (Baltimore). 2026 Oct 23;105(43):e51154. doi: 10.1097/MD.0000000000051154 (PMC13706495; doi:10.1097/MD.0000000000051154)
Supplement: Supplementary file 5 [file medi-105-e51154-s005.docx]

*Associations of Sleep Disturbance and Emotion Regulation With Perceived Workplace Safety and Fatigue Among Male Firefighters With Prior Occupational Accidents: A Cross-Sectional Study*

**Supplementary Table S3. PWSFS inter-item correlation matrix.**

| **Item** | **Q1** | **Q2** | **Q3** | **Q4** | **Q5** | **Q6** | **Q7** | **Q8** | **Q9** | **Q10** |
| --- | --- | --- | --- | --- | --- | --- | --- | --- | --- | --- |
| Q1 | 1.00 |  |  |  |  |  |  |  |  |  |
| Q2 | 0.47 | 1.00 |  |  |  |  |  |  |  |  |
| Q3 | 0.17 | 0.28 | 1.00 |  |  |  |  |  |  |  |
| Q4 | 0.34 | 0.22 | 0.08 | 1.00 |  |  |  |  |  |  |
| Q5 | 0.18 | 0.21 | 0.14 | 0.45 | 1.00 |  |  |  |  |  |
| Q6 | 0.24 | 0.11 | 0.33 | 0.05 | 0.03 | 1.00 |  |  |  |  |
| Q7 | 0.09 | 0.08 | 0.14 | 0.15 | −0.06 | 0.17 | 1.00 |  |  |  |
| Q8 | 0.80 | 0.41 | 0.19 | 0.37 | 0.21 | 0.15 | 0.19 | 1.00 |  |  |
| Q9 | 0.28 | 0.06 | 0.18 | 0.36 | 0.20 | 0.30 | 0.27 | 0.21 | 1.00 |  |
| Q10 | −0.06 | −0.01 | 0.21 | −0.06 | 0.13 | 0.17 | 0.14 | −0.13 | 0.20 | 1.00 |
| Note. N = 105. Pearson correlations among the ten PWSFS items. Q1 and Q8 correlate at 0.80 although they belong to different content areas; Q10 correlates weakly or negatively with most other items. | | | | | | | | | | |
