# Supplementary material for: Associations of sleep disturbance and emotion regulation with perceived workplace safety and fatigue among male firefighters with prior occupational accidents: A cross-sectional study
Source: Medicine (Baltimore). 2026 Oct 23;105(43):e51154. doi: 10.1097/MD.0000000000051154 (PMC13706495; doi:10.1097/MD.0000000000051154)
Supplement: Supplementary file 6 [file medi-105-e51154-s006.docx]

*Associations of Sleep Disturbance and Emotion Regulation With Perceived Workplace Safety and Fatigue Among Male Firefighters With Prior Occupational Accidents: A Cross-Sectional Study*

**Supplementary Table S5. Extended regression model with additional recorded covariates.**

| **Predictor** | **B** | **95% CI** | **β** | **P** |
| --- | --- | --- | --- | --- |
| Sleep disturbance (BaSIQS) | −1.833 | [−2.229, −1.438] | −.820 | < .001 |
| Sleep quality (SQS) | 0.726 | [−1.657, 3.108] | .047 | .547 |
| Emotion regulation (ERSQ) | −0.033 | [−0.128, 0.061] | −.052 | .486 |
| Age | 0.015 | [−0.324, 0.354] | .009 | .931 |
| Fire-service experience | −0.104 | [−0.465, 0.258] | −.059 | .570 |
| Current smoking | 0.734 | [−1.943, 3.410] | .033 | .588 |
| Monthly income | 0.364 | [−1.419, 2.147] | .025 | .686 |
| Marital status (married) | 4.463 | [0.163, 8.764] | .171 | .042 |
| Has children | −1.654 | [−5.899, 2.591] | −.071 | .441 |
| Rank (firefighter) | −1.772 | [−6.159, 2.614] | −.056 | .424 |
| Note. N = 105. R² = .687, adjusted R² = .654, F(10, 94) = 20.63, P < .001. The coefficient for sleep disturbance differs from the primary model by 1.3%. | | | | |
