# Supplementary material for: Associations of sleep disturbance and emotion regulation with perceived workplace safety and fatigue among male firefighters with prior occupational accidents: A cross-sectional study
Source: Medicine (Baltimore). 2026 Oct 23;105(43):e51154. doi: 10.1097/MD.0000000000051154 (PMC13706495; doi:10.1097/MD.0000000000051154)
Supplement: Supplementary file 7 [file medi-105-e51154-s007.docx]

*Associations of Sleep Disturbance and Emotion Regulation With Perceived Workplace Safety and Fatigue Among Male Firefighters With Prior Occupational Accidents: A Cross-Sectional Study*

**Supplementary Table S8. Influence- and specification-sensitive analyses of the primary model.**

| **Specification** | **B for sleep disturbance** | **95% CI or precision** | **R²** | **Note** |
| --- | --- | --- | --- | --- |
| Primary model (OLS) | −1.809 | [−2.170, −1.449] | .667 | Reference specification |
| HC3 robust standard errors | −1.809 | SE 0.200, t = −9.05 | .667 | Robust SE against OLS SE 0.182 |
| Deleting nine flagged observations | −1.922 | — | .731 | nine unique cases flagged by Cook D > 4/n or by leverage; the high-leverage case is among them; n = 96 |
| MM-estimator robust regression | −1.830 | — | — | Downweights outlying residuals |
| Leave-one-out refits | −1.889 to −1.743 | — | — | Range across all 105 refits |
| Sleep quality as indicator variables | −1.755 | — | .695 | No individual SQS level associated with the outcome |
| Note. N = 105 unless stated. All specifications retain sleep disturbance, sleep quality, emotion regulation, age and fire-service experience. The coefficient for sleep disturbance is stable in sign and magnitude across every specification. | | | | |
