# Supplementary material for: Associations of sleep disturbance and emotion regulation with perceived workplace safety and fatigue among male firefighters with prior occupational accidents: A cross-sectional study
Source: Medicine (Baltimore). 2026 Oct 23;105(43):e51154. doi: 10.1097/MD.0000000000051154 (PMC13706495; doi:10.1097/MD.0000000000051154)
Supplement: Supplementary file 8 [file medi-105-e51154-s008.docx]

*Associations of Sleep Disturbance and Emotion Regulation With Perceived Workplace Safety and Fatigue Among Male Firefighters With Prior Occupational Accidents: A Cross-Sectional Study*

**Supplementary Table S9. Cross-tabulation of PWSFS items Q1 and Q8.**

|  | **Q8 = 1** | **Q8 = 2** | **Q8 = 3** | **Q8 = 4** | **Q8 = 5** | **Total** |
| --- | --- | --- | --- | --- | --- | --- |
| Q1 = 1 | 2 | 0 | 1 | 1 | 0 | 4 |
| Q1 = 2 | 2 | 10 | 7 | 3 | 0 | 22 |
| Q1 = 3 | 1 | 2 | 28 | 1 | 0 | 32 |
| Q1 = 4 | 0 | 0 | 1 | 24 | 2 | 27 |
| Q1 = 5 | 0 | 0 | 2 | 0 | 18 | 20 |
| Total | 5 | 12 | 39 | 29 | 20 | 105 |
| Note. N = 105. Identical responses in 82 cases (78.1%); the two items differ in 23 cases, with differences from −3 to +2, so neither column duplicates the other. No respondent gave the same answer to all ten items, and every respondent used between two and five of the five response options. The next highest agreement between any two items is 49.5% (Q4 and Q5). | | | | | | |
