# Supplementary material for: Associations of sleep disturbance and emotion regulation with perceived workplace safety and fatigue among male firefighters with prior occupational accidents: A cross-sectional study
Source: Medicine (Baltimore). 2026 Oct 23;105(43):e51154. doi: 10.1097/MD.0000000000051154 (PMC13706495; doi:10.1097/MD.0000000000051154)
Supplement: Supplementary file 9 [file medi-105-e51154-s009.docx]

*Associations of Sleep Disturbance and Emotion Regulation With Perceived Workplace Safety and Fatigue Among Male Firefighters With Prior Occupational Accidents: A Cross-Sectional Study*

**Supplementary Table S4. Forced four-factor principal-axis analysis of the PWSFS items, with varimax rotation.**

| **Item** | **Assigned content area** | **Factor 1** | **Factor 2** | **Factor 3** | **Factor 4** | **h²** |
| --- | --- | --- | --- | --- | --- | --- |
| Q1 | Safety awareness | 0.85 | . | . | . | 0.78 |
| Q2 | Safety awareness | 0.52 | . | . | . | 0.34 |
| Q3 | Safety awareness | . | . | . | 0.62 | 0.43 |
| Q4 | Fatigue management | 0.30 | 0.50 | 0.32 | . | 0.45 |
| Q5 | Fatigue management | . | 0.88 | . | . | 0.81 |
| Q6 | Fatigue management | . | . | . | 0.43 | 0.29 |
| Q7 | Health monitoring | . | . | 0.34 | . | 0.15 |
| Q8 | Health monitoring | 0.86 | . | . | . | 0.79 |
| Q9 | Safety standards | . | . | 0.76 | . | 0.67 |
| Q10 | Safety standards | . | . | . | 0.41 | 0.23 |
| Note. N = 105. Principal-axis factoring with varimax rotation, four factors forced; loadings below \|0.30\| are shown as a period. Kaiser-Meyer-Olkin 0.64; Bartlett χ²(45) = 256.8, P < .001. Parallel analysis against 1000 random matrices retained two components (eigenvalues 2.93 and 1.55 against 95th-percentile random values of 1.66 and 1.46). The four factors account for 49% of the total variance. The rotated solution does not reproduce the assigned content areas: only Q4 and Q5 fall together within their own area, and Q4 loads on three factors. | | | | | | |
